# Outcomes of Induction Therapy in Patients With Acute Myeloid Leukemia During the COVID-19 Pandemic: A Retrospective Study From a Tertiary Cancer Center

**DOI:** 10.7759/cureus.29940

**Published:** 2022-10-05

**Authors:** Asif Iqbal, Nithin Raj Daniel, Raghavender Reddy, Munlima Hazarika, Roopam Deka

**Affiliations:** 1 Department of Medical Oncology, Dr B. Barooah Cancer Institute, Guwahati, IND; 2 Department of Oncopathology, Dr B. Barooah Cancer Institute, Guwahati, IND

**Keywords:** post-lockdown, coronavirus disease 2019, chemotherapy, aml, induction mortality, remission induction therapy, lockdown, covid-19

## Abstract

Background: The induction outcomes of patients with acute myeloid leukemia (AML) in India are at par with western data. But we fear that the absence of a robust defense mechanism during the COVID-19 pandemic and the resultant social, financial, political, and medical disturbance might have influenced outcomes. Hence, this study was conducted to establish relationships between the coronavirus disease 2019 (COVID-19) lockdown and induction treatment outcomes in AML patients.

Objective: To determine rates of induction remission, induction failure, and induction mortalities in patients with AML treated during the COVID-19 pandemic and compare those results between lockdown and post-lockdown periods in India.

Methods: This retrospective, observational study includes data from patients with AML who were started on induction therapy between May 1, 2020, and December 31, 2020. A total of 53 AML patients’ data was included in this study, divided into group 1 (n = 22) and group 2 (n = 31). Based on the COVID-19 pandemic-induced lockdown period in India, patients who were given induction therapy between May 1, 2020, and August 31, 2020, were included in Group 1 (Lockdown Phase group), and patients who were given induction therapy during the post-lockdown phase, i.e., September 1, 2020, to December 31, 2020, were included in Group 2 (Post-Lockdown Phase group). Data from AML patients of both sexes and all age groups were included. Data of patients who died before starting induction chemotherapy or patients who left the hospital before the completion of induction chemotherapy were excluded. Patients on induction therapy, be it intensive chemotherapy (ICT) or low dose chemotherapy (LCT), were included. Outcomes were analyzed after the first two induction cycles or 60 days of starting induction, whichever is earlier.

Results: The mean age of patients in Group 1 was 36.23±19.1 years and in Group 2 was 29±22.22 years; gender distribution was comparable in both groups. After the first induction, mortality in Group 1 was 36.36%, and in Group 2 was 45.16% (p = 0.036); partial remission in Group 1 and Group 2 was 50% and 29%, respectively (p = 0.036). Using survival analysis, death (event) after second induction was 149.77 days (111.1-188.5) in Group 1 and 137.23 (111.4-163.1) days in Group 2, which was statistically insignificant. Remission was achieved faster in Group 2, achieving complete remission in the mean of 94.96 days (74.5-115.5), while in Group 1, the mean of 147.18 days (110.9-183.5) (p = 0.034).

Conclusions: There was increased induction mortality and reduced complete remission (CR) during the post-lockdown phase despite the increased use of ICT, demonstrating an improvement in supportive care (availability of medicines, blood products). This shows that the improvement in supportive care did not show any change or improvement in the outcome for the patient. The mean days for remission were lower in the post-lockdown period compared to the lockdown phase, and patients who had achieved remission had a durable response.

## Introduction

Myelosuppression is an anticipated outcome of both leukemia and its treatment with chemotherapy. Hence, this needs careful monitoring, and most facilities are prepared for this treatment or disease-related consequence [1]. Acute myeloid leukemia (AML) constitutes a group of aggressive hematological malignancies. AML accounts for 70% of all acute leukemias in adults and 20% in children. In India, anecdotal reports from various cancer registries suggest that AML constitutes 1-2% of all cancer incidences [2,3]. Patients with AML present with features of bone marrow failure, typically with acute onset (<30 days), symptomatic anemia, fever, thrombocytopenic bleeding, etc.

The treatment of AML can be divided into intensive chemotherapy (ICT) and low-intensity chemotherapy (LCT). For the majority, ICT is preferred unless deemed unfit for it. The patients who fall into this unfit category include all patients older than 60 years or patients who, while younger than 60 years, were considered unfit by the attending physician. ICT for AML is divided into remission induction and post-remission therapy. Standard remission induction regimens for all AML subtypes, excluding acute promyelocytic leukemia (APL), include seven days of cytarabine infusion and three days of an anthracycline, commonly known as “7+3” chemotherapy. With this strategy, approximately 70-80% of adults younger than 60 years and 40-50% of older patients with good performance status achieve complete remission. The Cancer and Leukemia Group B (CALGB) established that three days of daunorubicin and seven days of cytarabine are more effective than two and five days, respectively, and that 10 days of cytarabine was not better than seven days. Also, 100 mg/m²/d of cytarabine is as effective as 200 mg/m²/d for seven days. Similarly, the dose of daunorubicin at 60 mg/m² was found to be superior to 45 mg/m² but was not inferior to 90 mg/m² in large cooperative trials. Achievement of transfusion independent state with platelet count > 1 lac/cumm, absolute neutrophil count (ANC) > 1000/cumm with less than 5% blasts in a bone marrow aspirate is considered as complete remission. Patients younger than 60 years, who have a significant residual disease without a hypocellular marrow on a day-14 bone marrow biopsy, should receive reinduction chemotherapy, either repeating “7+3” or using high dose Ara-C (high-dose cytarabine (HiDac))-based reinduction [4,5].

Looking into alternative induction therapies in patients between the ages of 60 and 75 years with therapy-related AML and AML with myelodysplasia-related changes, it is seen that CPX-351, a liposomal formulation of cytarabine/ daunorubicin in a fixed 5:1 molar ratio leads to an increased rate of complete remission and overall survival compared to standard 7+3 induction [6]. For medically-fit older patients with adverse cytogenetic/molecular features, treatment with either CPX-351 or hypomethylating agents (decitabine/azacytidine) can be offered. Decitabine or azacytidine provides a better balance of benefits and toxicities. They provide remissions in up to two-thirds of older patients with AML and can prolong survival compared to supportive care alone. In a study by Stone et al., for patients between the ages of 18 to 60 with an FLT3 mutation (internal tandem duplication (ITD or tyrosine kinase domain (TKD)), the addition of the multi-kinase inhibitor midostaurin on days 8-21 of 7+3 induction therapy and consolidation decreased the risk of death by 22% and increased overall survival at five years by 7%. This greatly improved from the outcomes studied with sorafenib (another tyrosine kinase inhibitor (TKI) with FLT3 inhibition) [7]. The CD33 antibody-drug conjugate gemtuzumab ozogamicin (GO) was recently re-approved by the FDA in combination with 7+3. Castaigne et al. found that this regimen improved event-free survival compared to 7+3 alone in patients with favorable and intermediate-risk AML [8].

Low-intensity treatment options can be offered for patients with favorable or intermediate prognostic features of AML who are less likely to tolerate intensive remission induction therapy. The selection of lower intensity agents should be individualized and is influenced by molecular features, availability of agents, physician’s experience, and patient preference. Like hypomethylating agents (decitabine, azacytidine), the hematologic toxicities are easily manageable, and these agents show hematologic improvement like blast clearance and transfusion independence after at least three cycles [9]. Similarly, low-dose cytarabine, glasdegib, venetoclax, and ivosidenib are new targeted molecules approved for elderly persons with AML [10]. These drugs are yet not available in the Indian market. Clofarabine is an acceptable option for patients of intermediate medical fitness with favorable or intermediate-risk categories who seek remission induction therapy. Still, in those for whom the intensive remission induction therapy may not be tolerable [11], single-agent GO is an acceptable remission induction therapy for selected older patients who are unable to tolerate intensive 7+3 therapy [12].

The coronavirus disease 2019 (COVID-19) pandemic has significant implications for blood transfusion. The shortage of blood bank inventory and unavailability of blood bank camps along with, shortages of voluntary blood donors due to travel restrictions, had a negative impact on the management of patients with acute leukemia, with increasing morbidities (symptomatic anemia, and thrombocytopenia) and induction mortalities [13,14]. In Assam (India), the COVID-19 pandemic could be essentially divided into two phases: the lockdown phase starting on March 23, 2020, to August 8, 2020, and the post-lockdown phase from September to December 2020. Travel restrictions were gradually lifted during August 2020.

This study has been undertaken to analyze the rates of induction mortality and remission in patients with AML treated in the lockdown and post-lockdown phases, respectively, and to compare the outcomes of both groups.

## Materials and methods

This is a retrospective, observational study of patients with AML treated between May 1, 2020, and December 31, 2020, in Dr. Bhubaneswar Barooah Cancer Institute, Guwahati, Assam, India. The study was conducted as per the Indian Council of Medical Research (ICMR) Guidelines for Biomedical Research on Human subjects, International Conference on Harmonisation - Good Clinical Practice (ICH GCP) Guidelines E6 (R1), Schedule Y (Amended Version 2013), and Declaration of Helsinki (Fortaleza, Brazil, October 2013). Approval was obtained from the Institutional Ethics Committee (IEC) of Dr. Bhubaneswar Barooah Cancer Institute (approval number: BBCI-TMC/SC/Appr/13/2021).

Patient selection

Inclusion Criteria

Patients with AML who started induction therapy (both ICT and LCT) between May 1 and December 31, 2020, were included. All age groups have been included in the present study with equal distribution of gender in both categories. No germline mutations were checked in these patients as it was during the lockdown period. 

Exclusion Criteria

Patients who died before starting induction chemotherapy and patients who left the hospital before the completion of induction chemotherapy were excluded from the study.

Study design and data collection

**Figure 1:**
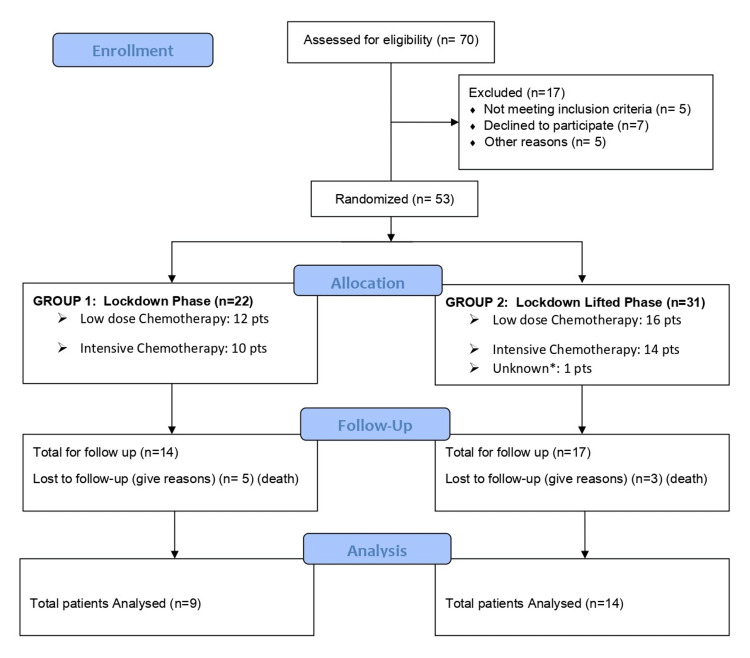
The CONSORT diagram showing the methodology of the study *This patient received azacytidine + venetoclax, a new combination at that time through an online prescription from Max Hospital, New Delhi. By some means, the drug was procured. This patient was excluded from the assessment. CONSORT: Consolidated Standards of Reporting Trials

The data of selected patients on outcomes of induction therapy (both ICT and LCT) were considered after the first two induction cycles or 60 days, whichever is earlier for both sets of patients (Figure 1). 

Patients were divided into two groups: (i) Group 1, which included patients who were given induction therapy between May 1 and August 31 (Lockdown Phase), and (ii) Group 2, which included patients who were given induction therapy between September 1 and December 31, 2020 (Post-Lockdown Phase). Clinical data management (CDM) was set up in the study database as per the data collection requirement. All data and records generated during this study were kept confidential on subject privacy, and their data and records were not used for any purpose other than conducting the study. Statistical analysis was done only after all entries were complete and data was locked. In this study, the bone marrow examination for remission status was done after two induction cycles in both ICT and LCT categories. 

Study endpoints and assessments

The primary endpoint was the proportion of patients with rates of induction remission, which was considered as an achievement of transfusion independent state with platelet count > 1 lakh/cumm, ANC > 1000/cumm with less than 5% blasts in a bone marrow aspirate. In the study, the bone marrow examination for remission status was done after two induction cycles in both ICT and LCT categories. After the first two cycles of chemotherapy and induction mortalities in patients with AML treated during the COVID-19 pandemic in 2020, the differences in outcomes during the lockdown phase and the post-lockdown phase were analyzed. The secondary endpoint was the median number of induction days in both groups., the median number of red blood cells (RBC), random donor plasma (RDP), or single-donor platelet (SDP) transfusions in both groups, and rates of infective induction deaths vs non-infective induction deaths in both groups. Failure to achieve complete remission after two induction cycles comes under the induction failure category. Deaths during induction therapy (within 60 days of starting induction) were not included in the induction failure. Decitabine or azacytidine provides a better balance of benefits and toxicities and was, therefore, used in LCT (in those patients who were not fit for ICT) in the patients participating in the study.

Statistical analysis

All data were summarized using descriptive analyses. Mean, standard deviation (SD), median, and range (min-max) were used to describe continuous variables. Frequency and percentage (two-sided 95%CI) were used to present categorical variables. The statistical analyses were performed using IBM SPSS Statistics for Windows, Version 27.0 (Released 2020; IBM Corp., Armonk, New York, United States).

## Results

Demographics and clinical characteristics

The mean age of patients in Group 1 was 36.23±19.1 years and in Group 2 was 29±22.22 years; gender distribution was comparable in both groups (Table 1)

**Table 1:** Patient demographics and other baseline variables *patients where category was not specified FAB: French–American–British; M0: undifferentiated acute myeloblastic leukemia; M1: acute myeloblastic leukemia with minimal maturation; M2: acute myeloblastic leukemia with maturation; M3: acute promyelocytic leukemia (APL); M4: acute myelomonocytic leukemia Group 1: lockdown phase; Group 2: post-lockdown phase

Baseline characteristics	Group 1 (n=22)	Group 2 (n=31)
Age (in years) (Mean + SD)	36.23 ± 19.1	29 ± 22.22
Gender (n (%))		
Female	11 (50%)	14 (45.8%)
Male	11 (50%)	17 (54.2%)
Blood Group (n (%))		
Unknown*	1 (4.5%)	10 (32.3%)
A+ve	6 (27.3%)	1 (3.2%)
AB+ve	2 (9.1%)	7 (22.6%)
B+ve	7 (31.8%)	1 (3.2%)
O-ve	1 (4.5%)	7 (22.6%)
O+ve	5 (22.7%)	5 (16.1%)
FAB Classification n (%)		
Not Classified*	5 (22.7%)	12 (38.7%)
M0	1 (4.5%)	1 (3.2%)
M1	3 (13.6%)	3 (9.7%)
M2	5 (22.7%)	11 (35.5%)
M3	2 (9.1%)	1 (3.2%)
M4	4 (18.2%)	3 (9.7%)
Prophylactic antifungal (n (%))		
No prophylactic antifungal	6 (27.2%)	5 (29.0%)
Fluconazole	1 (4.5%)	6 (19.4%)
Posaconazole	12 (54.5%)	9 (29.0%)
Voriconazole	3 (13.6%)	7 (22.6%)

Clinical Significance of the Baseline Characteristics

There is equal distribution of gender in both groups. Common blood group type in Group 1 (lockdown) is B+ve and A+ve, and in Group 2 (post-lockdown) is AB+ and O-ve. In the evaluation of patients according to the French-American-British (FAB) classification of AML, M2 (acute myeloblastic leukemia with maturation) was the most common class in both groups. Posaconazole was the most commonly used prophylactic antifungal in both groups followed by voriconazole.

Response to induction therapy

The proportion of patients receiving LCT was 54.5% and 51.6% in the lockdown and post-lockdown phases, respectively (Table 2).

**Table 2:** Proportion of patients undergoing ICT and LCT All values indicate n (%) other than those specified; *patients where the category was not specified ICT: intensive chemotherapy; LCT: low-dose chemotherapy Group 1: lockdown phase; Group 2: post-lockdown phase

Proportion of patients (n=53)	Group 2 (n=22)	Group 2 (n=31)	P-value (Chi-square)

Unknown*	-	1 (3.2)	0.7
ICT	10 (45.5)	14 (45.2)	
LCT	12 (54.5)	16 (51.6)	

**Figure 2:**
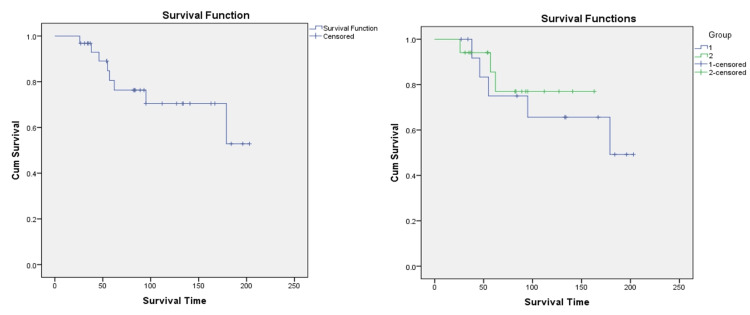
Kaplan-Meier survival curves of AML patients during lockdown (left) and post-lockdown (right) periods; Event: Death AML: acute myeloid leukemia

A significant difference was observed in response to remission induction therapy, with 40.9% in Group 1 and 25.8% in Group 2 achieving complete remission. Also, the mortality rate was higher in the post-lockdown period when compared to the lockdown period, and the difference was statistically significant (p-value 0.34) (Table 3) (Figure 2).

**Table 3:** Proportion of patients in different remission categories All values indicate n (%) other than those specified; **Statistically significant p-value < 0.05; *Patients who refused the treatment Group 1: lockdown phase; Group 2: post-lockdown phase

	Group 1 (n=22)	Group 2 (n=31)	P-value (Chi-square)
Mortality	8 (36.4%)	14 (45.2%)	0.034**
Complete Remission	9 (40.9%)	8 (25.8%)	
Failed Remission	5 (22.7%)	2 (6.5%)	
Refused*	0 (0.0%)	7 (22.6%)	

Also, 36.4% of patients in Group 1 and 45.2% of patients in Group 2 died due to various reasons like septicemia and bleeding, of which septicemia was the most common cause of death (Table 4). 

**Table 4:** Causes of death All values indicate n (%) or n other than those specified. *one patient had both septicemia and bleeding SARS CoV-2: severe acute respiratory syndrome coronavirus 2; CAD: coronary artery disease Group 1: lockdown phase; Group 2: post-lockdown phase

Cause of death	Group 1 (n=22)	Group 2 (n=31)	P-value (Chi-square)
Septicemia-related Deaths	9 (40.9%)	7 (22.6%)	0.290
Non-septicemia-related deaths	4 (18.18%)	10 (32.3%)	
Bleeding	2	2*	13
SARS CoV-2	0	2	17
CAD	1	1	
Others (aspiration, etc)	1	5	

The proportion and response of patients receiving at least two induction chemotherapy were comparable in both groups (Tables 5, 6).

**Table 5:** Proportion of patients with two rounds of induction chemotherapy All values indicate n (%) other than those specified Group 1: lockdown phase; Group 2: post-lockdown phase

Remission induction			
Proportion of patients (n=31)	Group 1 (n=14)	Group 2 (n=17)	P-value (Chi-square)
ICT	3 (37.5%)	6 (42.9%)	0.06
LCT	8 (62.5%)	14 (57.1%)	

**Table 6:** Response of patients after two rounds of induction chemotherapy All values indicate n (%) other than those specified Group 1: lockdown phase; Group 2: post-lockdown phase

	Group 1 (n=14)	Group 2 (n=17)	P-value (Chi-square)
Mortality	5 (35.7%)	3 (17.6%)	0.558
Complete remission	5 (35.7%)	3 (17.6%)	
Incomplete remission (partial remission + failed remission)	4 (28.5%)	11 (64.7%)	

If death was used as the event of interest, the mean survival days in the study population were 155.87 days. Both groups have comparable mean survival days (Table 7).

**Table 7:** Number of deaths and mean survival time AML: acute myeloid leukemia Group 1: lockdown phase; Group 2: post-lockdown phase

Group	N	Number of deaths	Mean survival time (in days) ± SE	P-value (Log rank)
Total AML patient	31	8	155.87 ± 13.77	-
Group 1	14	5	149.77 ± 19.74	0.6509
Group 2	17	3	137.230 ± 13.19	

**Figure 3:**
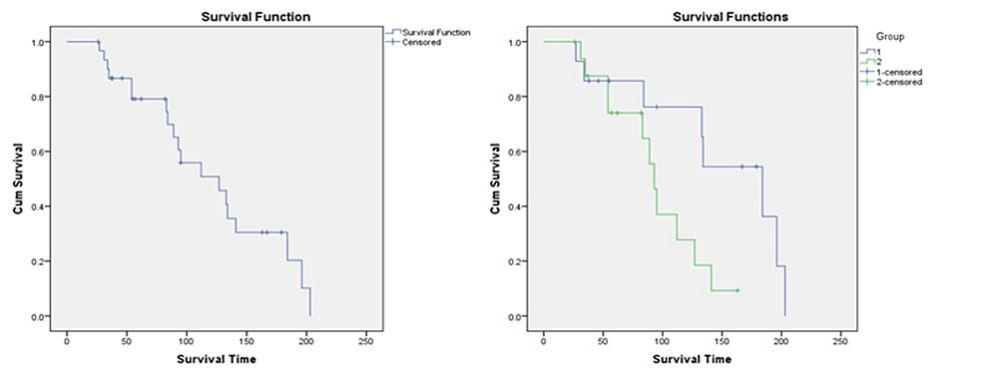
Kaplan Meier survival curves of AML patients during lockdown (left) and post-lockdown (right) periods; Event: Remission AML: acute myeloid leukemia

If remission was used as the event of interest, mean days for remission were lower in Group 2 (94.97 days), when compared to Group 1 (147.18 days), and the difference was statistically significant. (p-value 0.034) (Figure 3) (Table 8). The survival curves were well-separated from one another in Group 1 and Group 2.

**Table 8:** Number of remissions and mean survival time *Statistically significant p-value < 0.05 AML: acute myeloid leukemia Group 1: lockdown phase; Group 2: post-lockdown phase

Group	N	Number of remissions	Mean survival time (in days) ± SE	P-value (Log rank)
Total AML patient	31	19	121.03 ± 12.09	-
Group 1	14	8	147.18 ± 18.53	0.034*
Group 2	17	11	94.97 ± 10.47	

The median number of RBC, RDP, and SDP transfusions in Group 1 was 3, 11, and 0, respectively, and the median number of RBC, RDP, and SDP transfusions in Group 2 was 3, 12, and 0 respectively (Table 9).

**Table 9:** Median and mean of RBC, RDP, and SDP transfusions in both groups RBC: red blood cells; RDP: random donor plasma; SDP: single-donor platelet Group 1: lockdown phase; Group 2: post-lockdown phase

Transfusions	RBC	RDP	SDP
Total			
Mean ± SD	2.24 ± 1.8	10.02 ± 7.59	0.68 ± 1.15
Median	3.00	11.00	0.00
Group 1			
Mean ± SD	2.55 ± 1.73	10.59 ± 6.70	0.59 ± 1.14
Median	3.00	11.00	0.00
Group 2			
Mean ± SD	2.96 ± 1.77	12.00 ± 7.84	1.00 ± 1.28
Median	3.00	12.00	0.00

## Discussion

The COVID-19 infection started in China in early 2020 and, subsequently, in India, the number of cases began to rise somewhere around February/March 2000. Hence, the Government of India enforced a complete country-wide lockdown at the end of March 2020, similar to the other countries worldwide. This impacted patients with chronic or terminal diseases like AML, as they were unable to access healthcare facilities or had difficulty managing their treatment [15].

As the age of patients who are diagnosed with AML increases, especially ≥ 50 years, the proportion of favorable-risk group patients who received induction chemotherapy decreases rapidly [16]. So, the demographic data of this study is comparable to the existing results [17], although the sample size was small (n=51) (Table 1).

For many years, the FAB system has been used as the reference method for the classification of AML [1]. Majority of the participants in the present study in both groups, fall into the M2 (acute myeloblastic leukemia with maturation) category of the FAB classification. Other studies like the Khera et al. have also found similar data [18]. Globally, in the Japanese population also, M2 is a common subtype, while in the Australian population, M4 is a common subtype [19].

Myelosuppression is an anticipated outcome of both leukemia and its treatment with chemotherapy, so this needs careful monitoring, and most facilities are prepared for this treatment or disease-related consequence [1]. Common blood group type in Group 1 (lockdown phase) was B+ve and A+ve, and in Group 2 (post-lockdown phase), it was AB+ and O-ve. Within a few days of the start of the COVID-19 lockdown, blood banks in most states of India started facing an acute shortage of blood products, as blood donations dried up [20]. However, in this study, the median number of RBC, RDP, and SDP transfusions in both groups was comparable. Providing supportive RBC, RDP, or SDP transfusions is the backbone of leukemia care, and in the face of potential shortage and during a time when exposure to hospitals or public gatherings even for blood donation camps was limited, this led to modification in blood bank transfusion practices like randomized smaller camps, even reaching out to known donors for blood product collection [21].

In the initial phase of AML treatment, remission induction therapy is given to patients with a goal of decreasing leukemia cells to untraceable levels and thus re-establishing the production of normal blood cells, which was hampered due to the leukemic infiltration of bone marrow. But the intensive phase of AML treatment is associated with significant morbidity and mortality [22]. Studies in the past have demonstrated the role of low-dose cytarabine in AML, especially in elderly patients [23]. Patients who refuse intensive treatment or patients not suitable for 7+3 chemotherapy because of other comorbidities are offered lower-intensity treatments that can control their disease. In this study, 54.5% in Group 1 and 51.6% in Group 2 received LCT for remission induction therapy.

Looking into the Indian acute leukemia research database (INwARD) by Hematology Cancer Consortium (HCC), the induction mortality was found to be 16.9%, which ranges from 6.1% to 43% across different healthcare institutions or centers [24], which concurs with this study's data. Mortality was higher in Group 1 (45.2%) as compared to Group 2 (36.4%). AML induction-associated mortality rate in developed countries has been brought down to about 3-4%. However, in India, it continues to remain above 15%, especially during the COVID-19 pandemic, when added precautions were needed to reduce the risk of COVID-19 infection and related mortality.

Induction remission treatment of AML patients is followed by neutropenia, thrombocytopenia, etc., which lasts for a few weeks, during which there is an increased risk of infections, bleeding, and treatment-related mortality [24]. Major causes of mortality in the study cohort included septicemia, bleeding, and severe acute respiratory syndrome coronavirus 2 (SARS-CoV-2 infection. Septicemia was the most common cause of mortality in AML patients. In AML patients who are put on induction therapy, be it high-dose consolidation chemotherapy or low-dose consolidation chemotherapy, it leads to prolonged and serious granulocytopenia, increasing the risk of severe infections [25]. COVID-19 too is a factor in mortality. According to a study, 101 patients affected by hematological malignancy included 10 AML cases. Five patients (50%) died after a median of eight days and the deaths were COVID-19-related [26].

If death is used as the event of interest, the mean survival days in this study population was around 22 weeks (155.87 days). Some potent factors, including decreased performance status, comorbidities, duration of antecedent hematologic disorder (AHD), treatment outside the laminar flow room, and different compositions of cytogenetic risk are considered to correlate with worse outcomes of AML in conjunction with aging [27,28]. Treatment-related mortality is a major challenge in elderly and frail patients who receive induction chemotherapy [29].

In the present study, posaconazole was the most commonly used prophylactic antifungal, followed by voriconazole. In AML patients, there is a significant reduction in fungal-related mortality and documented invasive fungal infections (IFI), suggesting that antifungal prophylaxis should be given in patients after chemotherapy. Posaconazole has better results in this [30].

Complete response (CR) was higher in Group 1 (40.9%) when compared to Group 2 (25.8%), which are both lower than the INwARD database (52.8% CR) [24].

If remission was used as the event of interest, mean days for remission were lower in Group 2 (94.97 days), when compared to Group 1 (147.18 days), and the difference was statistically significant. In a 121-patient study, a four-year event-free survival (EFS) rate of 35.8% (95%CI, 28-46%) and a median EFS time of 19 months were seen. Nine patients did not survive induction [3].

COVID-19 resulted in many casualties not just because of its pathologies but also due to its social implication. Acute leukemia patients are at a greater risk of severe complications, as the presenting symptoms can be similar; healthcare practitioners should imperatively keep the possibility of acute leukemia in mind. However, physicians should be aware of their interactions with other drugs used to treat SARS-CoV-2-related infections/complications, such as antibiotics, anti-viral drugs, and various other drugs that prolong QTc or impact targeted-therapy pharmacokinetics [31].

The limitation of the present study is its retrospective nature and the study design, which did not allow a direct comparison.

## Conclusions

M2 was the most common class of AML, and LCT was received by 54.5% in Group 1 (lockdown phase) and 51.6% in Group 2 (post-lockdown phase). A total of 40.9% in Group 1 and 25.8% in Group 2 achieved complete remission. A total of 35.7% in Group 1 and 17.6% in Group 2 achieved complete remission after two cycles of induction chemotherapy. The mortality rate during the remission induction therapy period was higher during the post-lockdown period, with septicemia being the most common cause of death. The mean days for remission were lower in the post-lockdown period (94.97 days), compared to the lockdown phase (147.18 days). The median number of RBC, RDP, and SDP transfusions in the lockdown phase was 3, 11, and 0, respectively, and in the post-lockdown phase was 3, 12, and 0, respectively. Posaconazole was the most used prophylactic antifungal agent.

There was increased induction mortality and reduced complete remission (CR) during the post-lockdown phase despite the increased use of ICT, demonstrating an improvement in supportive care (availability of medicines, blood products), which showed that the improvement in supportive care did not show any change or improvement in the outcome for the patient. This study can underline the importance of supportive care, which affects outcomes directly and indirectly by undermining physicians’ decisions regarding the choice of therapy.

## References

[1] (2022). American Cancer Society. Key Statistics for Acute Myeloid Leukemia. http://www.cancer.org/cancer/acute-myeloid-leukemia/about/key-statistics.html..

[2] Asthana S, Labani S, Mehrana S, Bakhshi S (2018). Incidence of childhood leukemia and lymphoma in India. Pediatr Hematol Oncol J.

[3] Jain H, Rengaraj K, Sharma V (2020). Infection prevalence in adolescents and adults with acute myeloid leukemia treated in an Indian tertiary care center.. JCO Global Oncology.

[4] Preisler H, Davis RB, Kirshner J (1987). Comparison of three remission induction regimens and two postinduction strategies for the treatment of acute nonlymphocytic leukemia: a cancer and leukemia group B study. Blood.

[5] Rai K, Holland J, Glidewell O (1981). Treatment of acute myelocytic leukemia: a study by cancer and leukemia group B. Blood.

[6] Lancet JE, Cortes JE, Hogge DE (2014). Phase 2 trial of CPX-351, a fixed 5:1 molar ratio of cytarabine/daunorubicin, vs cytarabine/daunorubicin in older adults with untreated AML. Blood.

[7] Stone RM, Mandrekar SJ, Sanford BL (2017). Midostaurin plus chemotherapy for acute myeloid leukemia with a FLT3 mutation. N Engl J Med.

[8] Castaigne S, Pautas C, Terré C (2012). Effect of gemtuzumab ozogamicin on survival of adult patients with de-novo acute myeloid leukaemia (ALFA- 0701): a andomized, open-label, phase 3 study. Lancet.

[9] Wang ES (2014). Treating acute myeloid leukemia in older adults. Hematology Am Soc Hematol Educ Program.

[10] (2022). FDA. Daunorubicin and Cytarabine prescribing information [Internet]. [cited. VYXEOS™ (daunorubicin and cytarabine) liposome for injection, for intravenous use (Label).

[11] Hesketh PJ, Kris MG, Basch E (2017). Antiemetics: American Society of Clinical Oncology clinical practice guideline update. J Clin Oncol.

[12] Amadori S, Suciu S, Selleslag D (2016). Gemtuzumab ozogamicin versus best supportive care in older patients with newly diagnosed acute myeloid leukemia unsuitable for intensive chemotherapy: results of the randomized phase III EORTC-GIMEMA AML-19 trial. J Clin Oncol.

[13] Stanworth SJ, New HV, Apelseth TO (2020). Effects of the COVID-19 pandemic on supply and use of blood for transfusion. Lancet Haematol.

[14] Al Mahmasani L, Hodroj MH, Finianos A, Taher A (2021). COVID-19 pandemic and transfusion medicine: the worldwide challenge and its implications. Ann Hematol.

[15] Kretchy IA, Asiedu-Danso M, Kretchy JP (2021). Medication management and adherence during the COVID-19 pandemic: Perspectives and experiences from low-and middle-income countries. Res Social Adm Pharm.

[16] Yoo KH, Kim HJ, Min YH (2021). Age and remission induction therapy for acute myeloid leukemia: an analysis of data from the Korean acute myeloid leukemia registry. PLoS One.

[17] Demichelis-Gómez R, Alvarado-Ibarra M, Vasquez-Chávez J (2021). Treating acute leukemia during the COVID-19 pandemic in an environment with limited resources: a multicenter experience in four Latin American countries. JCO Glob Oncol.

[18] Khera R, Ahmed F, Mundada MC (2017). Multiplex approach in classification, diagnosis, and prognostication in acute myeloid leukemia: an experience from tertiary cancer center in South India. Indian J Med Paediatr Oncol.

[19] Nakase K, Bradstock K, Sartor M (2000). Geographic heterogeneity of cellular characteristics of acute myeloid leukemia: a comparative study of Australian and Japanese adult cases. Leukemia.

[20] (2022). Covid-19: Lockdown creates acute shortage at blood banks. https://timesofindia.indiatimes.com/india/covid-19-lockdown-creates-acute-shortage-at-blood-banks/articleshow/74958205.cms.

[21] Wilde L, Isidori A, Keiffer G, Palmisiano N, Kasner M (2020). Caring for AML patients during the COVID-19 crisis: an American and Italian experience. Front Oncol.

[22] (2022). PDQ Cancer Information Summaries [Internet].

[23] Castaigne S, Tilly H, Sigaux F, Daniel MT, Degos L (1985). Treatment of leukemia with low dose Ara-C: a study of 159 cases. Haematol Blood Transfus.

[24] Kayal S, Sengar M, Jain H (2019). Induction related mortality in acute myeloid leukemia multivariate model of predictive score from the Indian Acute Leukemia Research Database (INwARD) of the Hematology Cancer Consortium (HCC). Blood.

[25] Ferrara F, Schiffer CA (2013). Acute myeloid leukaemia in adults. Lancet.

[26] Ferrara F, Zappasodi P, Roncoroni E, Borlenghi E, Rossi G (2020). Impact of Covid-19 on the treatment of acute myeloid leukemia. Leukemia.

[27] Walter RB, Estey EH (2015). Management of older or unfit patients with acute myeloid leukemia. Leukemia.

[28] van der Holt B, Breems DA, Berna Beverloo H, van den Berg E, Burnett AK, Sonneveld P, Löwenberg B (2007). Various distinctive cytogenetic abnormalities in patients with acute myeloid leukaemia aged 60 years and older express adverse prognostic value: results from a prospective clinical trial. Br J Haematol.

[29] Stone RM, Berg DT, George SL (1995). Granulocyte-macrophage colony-stimulating factor after initial chemotherapy for elderly patients with primary acute myelogenous leukemia. Cancer and Leukemia Group B. N Engl J Med.

[30] Robenshtok E, Gafter-Gvili A, Goldberg E, Weinberger M, Yeshurun M, Leibovici L, Paul M (2007). Antifungal prophylaxis in cancer patients after chemotherapy or hematopoietic stem-cell transplantation: systematic review and meta-analysis. J Clin Oncol.

[31] Gavillet M, Carr Klappert J, Spertini O, Blum S (2020). Acute leukemia in the time of COVID-19. Leuk Res.

