# Correction: Outcomes of Induction Therapy in Patients With Acute Myeloid Leukemia During the COVID-19 Pandemic: A Retrospective Study From a Tertiary Cancer Center

**DOI:** 10.7759/cureus.c81

**Published:** 2022-11-17

**Authors:** Asif Iqbal, Nithin Raj Daniel, Raghavender Reddy, Munlima Hazarika, Roopam Deka

**Affiliations:** 1 Department of Medical Oncology, Dr B. Barooah Cancer Institute, Guwahati, IND; 2 Department of Oncopathology, Dr B. Barooah Cancer Institute, Guwahati, IND

This article has been corrected to include the following co-authors which were erroneously omitted by the submitting author, Asif Iqbal, prior to publication. Dr. Iqbal deeply regrets this error and apologizes for the confusion and inconvenience.

- Nithin Raj Daniel
- Raghavender Reddy
- Munlima Hazarika
- Roopam Deka

